# Ultraviolet Light Enhances the Bovine Serum Albumin Fixation for Acid Fast Bacilli Stain

**DOI:** 10.1371/journal.pone.0089370

**Published:** 2014-02-21

**Authors:** Jun-Ren Sun, Yun-Hsiang Cheng, Pei-Yin Lai, Shih-Yi Lee, Yu-Ching Chou, Yung-Chieh Fu, Chen-Cheng Wu, Tzong-Shi Chiueh

**Affiliations:** 1 Graduate Institute of Medical Science, National Defense Medical Center, Taipei, Taiwan; 2 Division of Clinical Pathology, Department of Pathology, Tri-Service General Hospital and National Defense Medical Center, Taipei, Taiwan; 3 Division of Clinical Microbiology, Department of Pathology and Laboratory Medicine, Taipei Veterans General Hospital, Taipei, Taiwan; 4 School of Public Health, National Defense Medical Center, Taipei, Taiwan; National Institute for Agriculture and Veterinary Research, IP (INIAV, I.P.), Portugal

## Abstract

The use of a liquid culture system such as MGIT broth has greatly improved the sensitivity of isolating mycobacteria in clinical laboratories. Microscopic visualization of acid fast bacilli (AFB) in the culture positive MGIT broth remains the first routine step for rapidly indicating the presence of mycobacteria. We modified an ultraviolet (UV) light fixation process to increase AFB cells adherence to the slide. The retained haze proportion of a 1-cm circle marked area on the smear slide was quantified after the staining procedure indicating the adherence degree of AFB cells. More AFB cells were preserved on the slide after exposure to UV light of either germicidal lamp or UV crosslinker in a time-dependent manner. We demonstrated both the bovine serum albumin (BSA) in MGIT media and UV light exposure were required for enhancing fixation of AFB cells. While applying to AFB stains for 302 AFB positive MGIT broths in clinics, more AFB cells were retained and observed on smear slides prepared by the modified fixation procedure rather than by the conventional method. The modified fixation procedure was thus recommended for improving the sensitivity of microscopic diagnosis of AFB cells in culture positive MGIT broth.

## Introduction

Tuberculosis (TB) continues to be a tremendous public health threat and remains the leading infectious cause of death [1]. Rapid diagnosis is critical for initiation of clinical treatment and infection control to prevent further transmission of tuberculosis [2], [3]. Because MTB grows faster in broth than on solid media, the use of broth based system is usually the recommended practice for faster isolation and improved test sensitivity [4]. The introduction of the automated broth based system, such as Bactec MB9000, Bactec MGIT 960, BacT/Alert and ESP Culture System with continuous monitoring shortens the turnaround time and decreases the workload of the laboratory [5], [6]. Acid fast bacilli (AFB) microscopy from culture positive broth typically serves as the initial step for discriminating between mycobacteria and contaminating microbes. Heat fixation is widely used to ensure the adherence of a bacterial smear onto a glass slide for the subsequent stain processes [3], [7]. However, heat fixation is not efficient enough to avoid AFB cells falling off the slide, and thus limits the sensitivity of microscopic observation [8].

Ultraviolet (UV) light is defined as light with shorter wavelength between the electromagnetic spectrum of X rays and visible light [9]. The use of UV light in biological safety cabinet (BSC) has a long history and it is moderately effective for decontaminating work surfaces and killing airborne microorganisms [10], [11]. A recent study indicated that the slides fixed overnight by UV light within a BSC can improve sensitivity of AFB staining in clinical specimens [8]. In our laboratory, the UV fixation enhancing effect was also found in AFB staining of the culture positive MGIT smear. However, the detail mechanism regarding how the UV light enhances the fixation of AFB cells on the slide remains unknown. The present study is aimed to verify and dissect the parameters related to the UV fixation enhancing effect.

## Materials and Methods

### Reference Strains

All reference strains used in this study were collected from the American Type Culture Collection (ATCC, Manassas, VA), including *Mycobacterium tuberculosis* ATCC27294, *Mycobacterium fortuitum* ATCC6481, *Mycobacterium kansasii* ATCC12478, *Mycobacterium abscessus* ATCC19977 and *Mycobacterium avium* ATCC25291.

### Clinical Specimen Processing

All clinical specimens from patients were processed and decontaminated within 24 h after collection, as described previously [12], [13]. A mycobacteria growth indicator tube (MGIT) culture medium that yielded a bacterial growth signal using the MGIT 960 system (Becton Dickinson, Franklin Lakes, NJ, USA) was to be further confirmed for AFB by microscopic observation. All of the AFB positive MGIT broths in this study were identified as MTB complex using the MGIT TBc Identification Test (TBc ID; Becton Dickinson), as described by the manufacturer [14]. An aliquot of the positive MGIT culture was also inoculated onto Lowenstein–Jensen (LJ) slants (Becton Dickinson) for further identification. The clinical NTM isolates were identified to the species level by DNA sequencing of the partial- length 16S rRNA gene [13], [14].

### Quantification of the Fixation Efficiency

We used the germicidal lamp in BSC or the spectrolinker XL-1000 UV crosslinker (Spectronics,Westbury, NY) for the UV exposure experiment. The following were purchased from Becton Dickson: 1. MGIT tube containing 7 mL of modified Middlebrook 7H9 broth base; 2. PANTA antibiotic mixture containing a lyophilized mixture of antimicrobial agents; 3. OADC (oleic acid- albumin- dextrose- catalase) enrichment containing 15 mL Middlebrook OADC enrichment. PANTA antibiotic mixture was dissolved in 15 mL OADC enrichment to generate the supplement solution. Prior to inoculating the MGIT liquid media with the specimen, 0.8 mL supplement solution was added into each MGIT tube to generate MGIT working media. Middlebrook 7H9 broth, OADC was also purchased from Becton Dickinson. Bovine serum albumin (BSA) was purchased from Sigma Aldrich (Sigma-Aldrich, Milwaukee, WI) and was prepared at 50 g/L in H_2_O as working solution. MGIT broth media was aspirated and transferred 2 drops onto a pair of slides. Each drop was then spread out to make a thin film circled by a crayon line about 1-cm in diameter. All slides were air dried and heat fixed on an electric slide warmer (65°C, 2 hours) in BSC. After heat fixation, only one of each slide pair was exposed to UV light (UV-C [100 to 280 nm]) within a type IIA biological safety cabinet (Thermo scientific class 2A) for various periods (0, 2, 4, 8 and 16 hours). The other slide of each pair was masked by dark paper as control. The energy output of the UV bulbs was approximately 3500 µW/cm^2^ per second in UV crosslinker. Subsequently, slides were stained using Acid-Fast Bacilli stain Kits (Kinyoun stain or Ziehl-Neelsen stain). The Acid-Fast Bacilli stain Kits was purchased from Becton Dickinson (Becton Dickinson, Franklin Lakes, NJ, USA) and used according to the manufacturer’s recommendation. Each test was performed in triplicate, and all results were photographed by the UVP Biospectrum AC imaging system (UVP, LLC). In order to quantify the fixation efficiency, all images were processed using the Image J software (Image J, National Institutes of Health, Bethesda, MD) for quantifying the dark intensity in each circle area of the slides. All of the data were displayed as mean ± SEM (standard error of the mean).

### Prospective Clinical Evaluation for the Efficacy of UV Enhanced Fixation

To assess the clinical performance of the modified fixation process for detecting AFB cells, a total of 302 culture tubes reported with mycobacteria by the routine identification process from individual patients were included in this analysis. A pair of slides was prepared as described above for each selected culture tube. All slides were randomly numbered to ensure they were examined without foreknowing their fixation method. The same senior technician prepared and stained all the slides to minimize the variation before microscopic examination. Each slide was interpreted by three medical technologists independently at two different laboratories in Tri-Service General Hospital and Taipei Veterans General Hospital. The total numbers of AFBs in 100 high-power visual fields were counted at 1000X magnification using an Olympus CX31 light microscope. The smears were interpreted and reported by the stratified scale as following: negative, none visible/100 visual fields; scanty positive, 1–9/100 visual fields; 1+, 10–99/100 visual fields; 2+, 100–999/100 visual fields; and 3+, >1, 000/100 visual fields).

### Data Analysis

Statistical analysis was carried out using SPSS version 20.0 software package; calculation of statistical difference between various groups was based on the Mann-Whitney U test. Differences were considered statistically significant when P<0.05.

## Results

### Additional UV Exposure Enhances the Conventional Heat Fixation for AFB Stain

**Figure 1:**
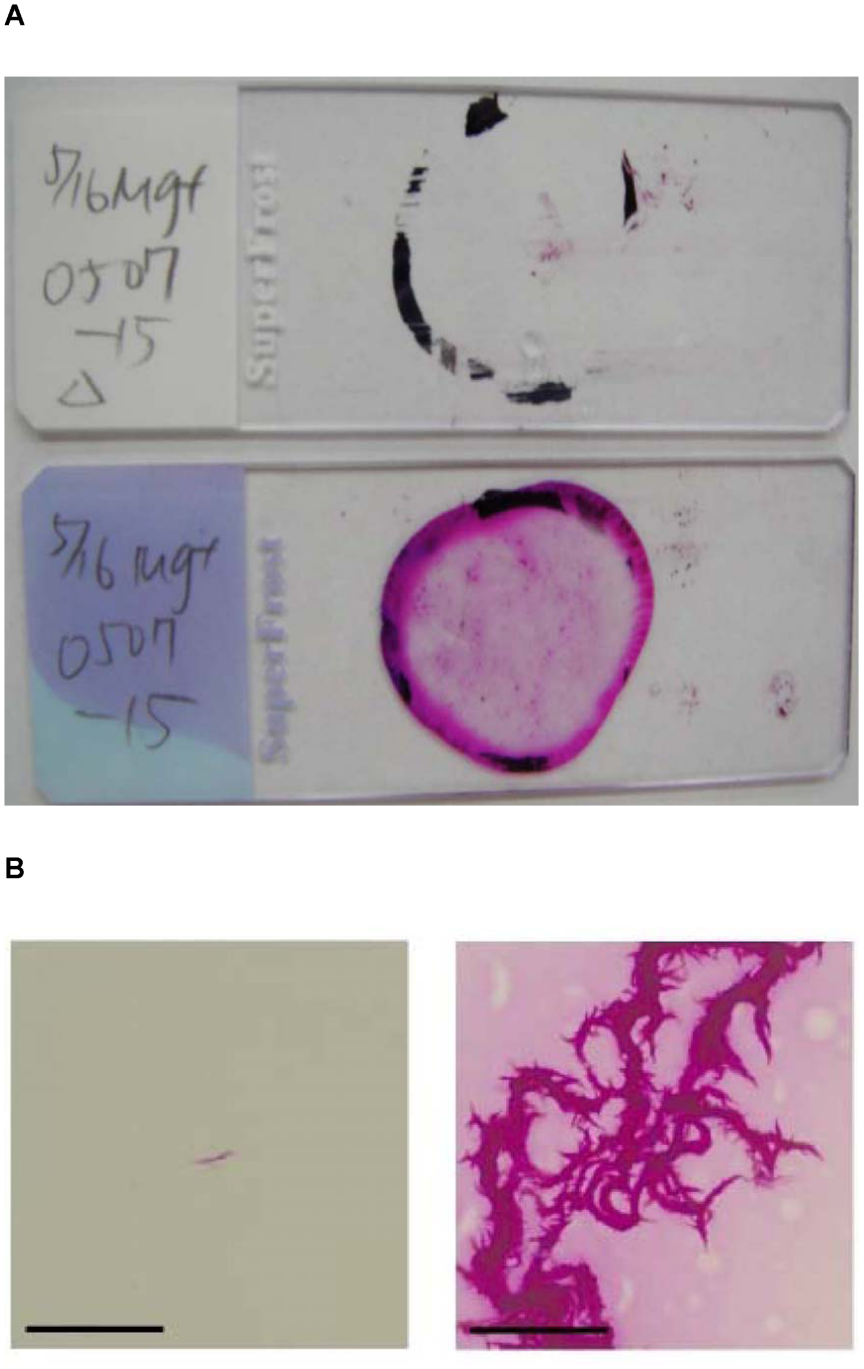
UV exposure effect on AFB smear slides. (A) Gross observation of AFB smear slides fixed by the conventional method (top) and by additional UV exposure (bottom). Haze crayon circled area was observed on the UV exposed slide. (B) Apparent cord form AFB cells were observed microscopically in slides fixed with the modified UV fixation process (right); rare cord form AFB cells were found in slides fixed with the conventional process (left). Scale bar, 50 µm.

The analytical effect of the UV exposure was tested with a panel of mycobacterial reference strains. One drop of positive growth MGIT broth with reference strains was deposited onto one pair of slides. Subsequently, one slide was exposed to UV light within a BSC. The other slide was masked by dark paper as control within a BSC. When the AFB staining was done, thick dark blotch were obtained in all mycobacterial strains after UV exposure but not in control. The slide after UV exposure could generate thick dark blotch which showed significantly more AFB on slide among the 5 species. We found the increase in haze of thick blotch is associated with more AFB cells present on the slide either Kinyoun stain or Ziehl-Neelsen stain (data not show). Pair of slides was collected and compared for their appearance grossly (Figure 1A) and microscopic finding (Figure 1B) after AFB staining. The slide fixed by the conventional process mostly showed a clean area in the crayon circle of the slide. In contrast, the crayon circle area on its counter slide fixed by the additional UV exposure by germicidal lamp within a BSC mostly remained cloudy or haze. Besides the gross observation, the typical microscopic cord form of MTB was observed easily in the UV fixation enhancing slides. The conventionally fixed slides could only show sparsely distributed AFB under microscope. Both gross and microscopic observation demonstrated that the additional UV exposure by UV light within a BSC could increase in haze of thick blotch which was associated with more AFB cells adhering to the slide through the staining procedures.

### UV Exposure Increases Coating the MGIT Working Media on Slides

**Figure 2:**
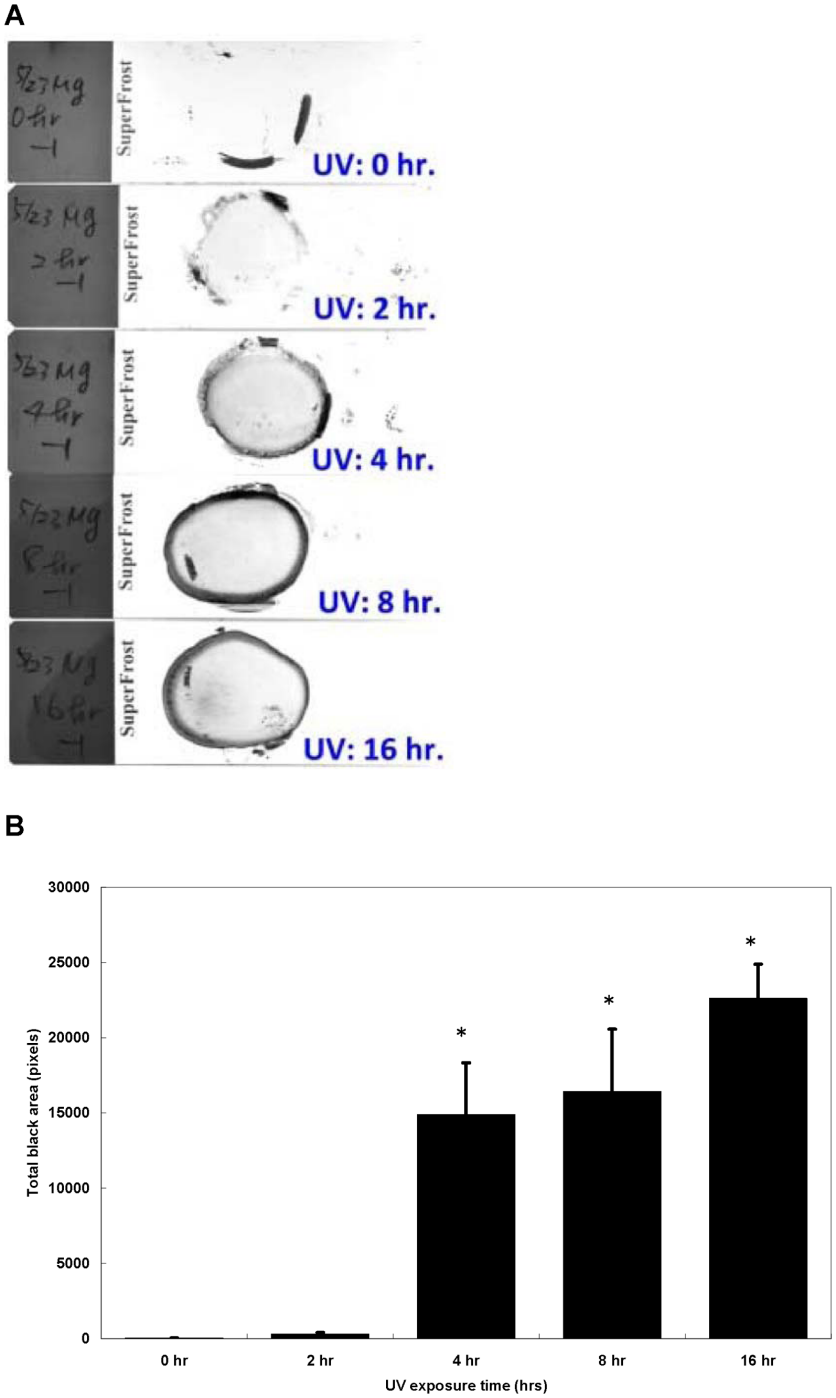
Additional UV exposure by UV light within a BSC increases haze of the crayon circled area on the AFB smear slides. (A) UV exposure by UV light within a BSC enhances MGIT working medium coating on slides in a time-dependent manner. (B) Quantify total pixels of the crayon circled area on the digital photo of the slide using Image J software. Each sample was tested in triplicate. Each bar represents the average of results from three independent experiments, and the error bars represent two standard deviations. Statistical significance was analyzed using Mann-Whitney test (* p<0.05).

To confirm the fixation effect of UV light treatment by germicidal lamp within a BSC, MGIT working media without bacteria cells were smeared and treated by UV irradiation of the germicidal lamp within a BSC for various periods of time. The longer time of UV exposure, the thicker dark blotch were retained in the crayon circled area on the slide (Figure 2A). By quantifying total pixels of the crayon circled area on the digital photo of the slide, there were a significant increase in the total pixels of the crayon circled area on the slide at least four hours of UV irradiation (Mann-Whitney Test; P value <0.05) (Figure 2B). This data suggested that some components in the MGIT working media might be responsible for the coating effect enhanced by UV light exposure.

### BSA Increases Coating on Slides after UV Exposure in a Time-dependent Manner

**Figure 3:**
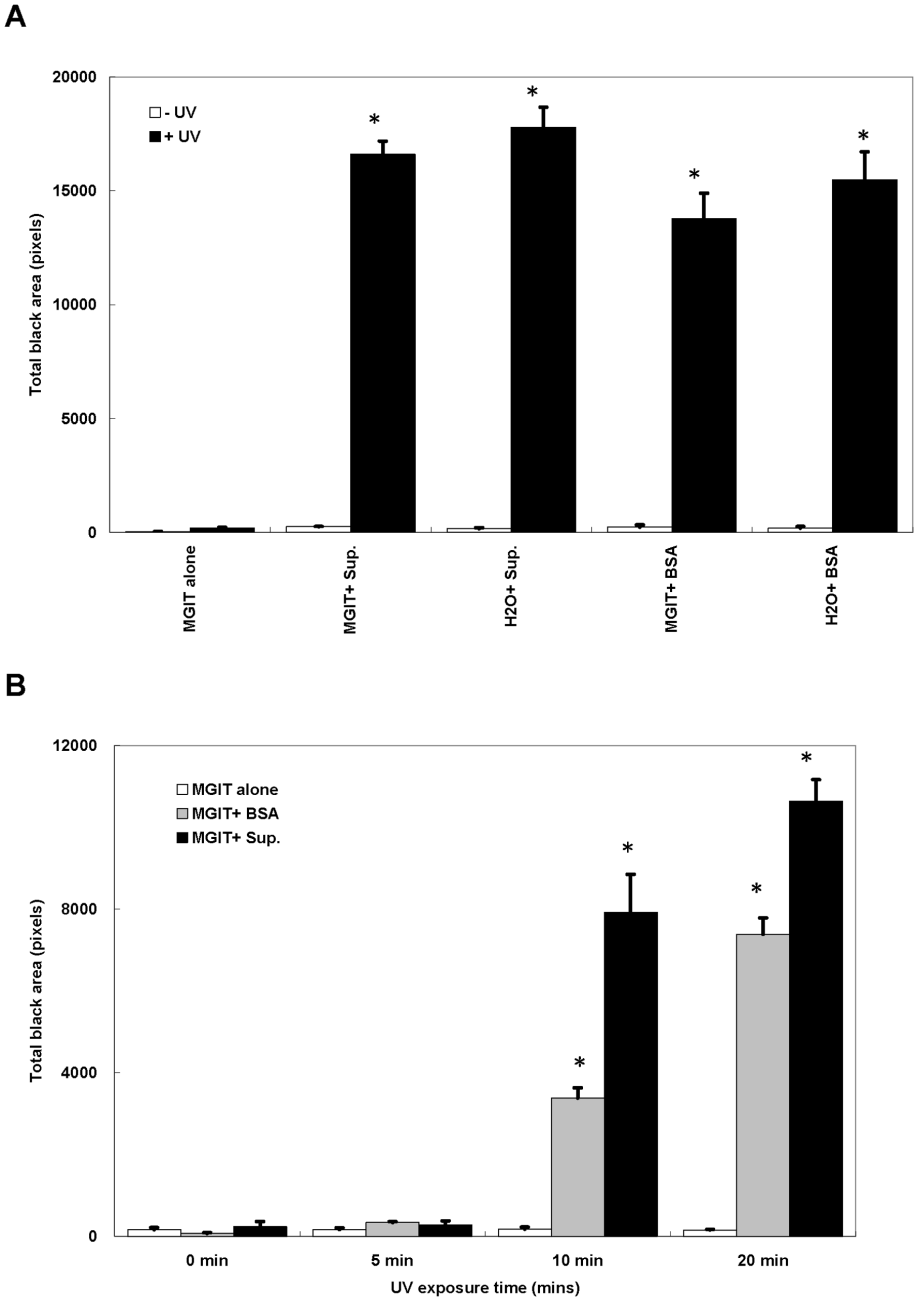
Both BSA and UV exposure are required for increasing digital pixels of the crayon circled area on the photo of AFB slide. (A) MGIT media alone (MGIT alone), MGIT media with supplement solution (MGIT+Sup.), distilled water with supplement solution (H_2_O+ Sup.), MGIT media with BSA (MGIT+ BSA) and distilled water with BSA (H_2_O+BSA) were smeared on slides with (white bars) or without (black bars) UV exposure by UV light within a BSC, respectively. (B) MGIT media alone (white bars), MGIT media with BSA (gray bars) and MGIT media with supplement (black bars) were smeared on slides and were exposed to UV of various time by UV light within a UV crosslinker, respectively. Each sample was tested in triplicate. Each bar represents the average of results from three independent experiments, and the error bars represent two standard deviations. Statistical significance was analyzed using Mann-Whitney test (* p<0.05).

MGIT base media, supplement solution, and BSA were tested separately for their UV-enhanced fixation effect by UV light within a BSC (Figure 3A). The total pixels of the crayon circled area increased significantly only in the presence of supplement solution or BSA (50 µg/L) with MGIT base media or distilled water (Mann-Whitney Test; P value <0.05). BSA was therefore the determinant component for the UV-enhanced fixation. Another UV light source by germicidal lamp within a spectrolinker UV crosslinker was applied to test the minimal energy for successfully enhancing fixation (Figure 3B). Superior to the germicidal UV lamp in BSC, it only took 10-minute exposure for the UV crosslinker to result in the same fixation enhancing effect (Mann-Whitney Test; P value <0.05). The data suggested that BSA with UV exposure improves firm slide coating for AFB stain with either of UV sources.

### Verify the Clinical Performance of the Modified Fixation Process Prospectively

We further evaluated the performance of the modified fixation process in our routine service prospectively. A total of 302 positive growth MGIT broths were included in this study from March 2011 to July 2012. They had been confirmed with diagnosis of mycobacterium after 4–6 weeks routine identification processes. All specimens were smeared on two slides that were fixed with the conventional (−UV) or modified (+UV) process. A total of 302 slide pairs were evaluated independently at 2 different laboratories by three medical technologists. The stratified scale results of microscopic observation (n = 1812) were recorded in Table 1. AFB cells could be found microscopically in all the slides fixed with the modified process. However, 308 slides were falsely reported as “negative” while fixed by the conventional process. The number of “2+ or 3+” positive smear in the modified fixation slides (n = 784) was significantly higher than in the conventionally fixed slides (n = 168). And the number of “scanty+ or 1+” positive smear in the modified fixation slides (n = 122) was significantly lower than in the conventionally fixed slides (n = 430). The stratified scales of AFB cells count were significantly higher in the modified fixation slides than in the conventionally fixed slides (McNemar test; P value <0.05).

**Table 1:** Stratified scale reports of AFB cells observed over 302 confirmed clinical positive growth MGIT broths with AFB cells.

	AFB smears, n			
Stratified scale	− UV	+ UV	Total	P value
2+ or 3+	168	784	952	<0.0001
scanty or 1+	430	122	552	
negative	308	0	308	

The AFB positive MGIT broth was smeared on two slides that were fixed with the conventional (−UV) or modified fixation process (+UV). All slides were interpreted by three medical technologists. Statistical significance was analyzed using McNemar test.

## Discussion

Smear and staining the bacterial cells on glass slides are basic laboratory techniques of microbiology. Some evidence in Gram stain suggests the fixation of bacterial cells to the glass slide prior to staining may influence the final stain results [7], [10]. Both physical and chemical fixation methods have been applied before staining to prevent AFB cells from falling off during staining. The most common physical method is heat fixation. It is done by passing the bottom of a slide through the tip of the burner flame for several times or using an electric slide warmer. Methanol and ethanol are the most common fixative chemicals [10], [15]. A number of studies have shown that chemical fixation gives more reliable Gram staining results than heat fixation, and it has even been recommended as a standard staining method for automated instruments [15], [16]. However, heat fixation is rather preferred in laboratories because of its simplicity and safety. In the present study, we modified the heat fixation process with additional UV exposure. The number of AFB cells retained was significantly higher on the slide with the modified fixation process.

A recently study described that a thicker dry blotch of BacT/Alert MP medium was shown in Auramine O staining after fixation with UV light overnight [8]. The sensitivity of microscopy therefore increased to 82%. However, previous studies have demonstrated that the exposure of UV light, such as sun light or germicidal lamp in a BSC, results in decreased stain ability for acid fast or Gram stain [17]–[20]. The UV light was believed to destroy the permeability or structure of the cell membrane and make mycobacteria less acid-fast. The guideline of acid fast stain therefore recommends avoidance of UV exposure. In this study, the similar enhance effect of UV light fixation was found as recent publication, although the high concentration of more than 10^6^/mL AFB bacteria in MGIT positive tube may conceal the interference effect of UV exposure. We suggest the mechanism in the recent publication was similar to our finding because the formulation of BacT/Alert MP medium also includes BSA (0.5% w/v). The UV fixation effect might be attributed to two proposed steps. First, AFB cells were covered with BSA onto the surface of slides compactly. Second, UV light provided high energy to denature BSA and bacteria, thereby trigger cross-linking with the slide surface.

UV irradiation is a high energy source for cross-linking materials including cross-link of polymer in synthetic polymer chemistry, patterning of protein or nucleotide on glass surface in the biological sciences [21]–[23]. An application of BSA is used as a coat protein on the cover slide for cell attachment [24]. In this study, the enhanced effect by UV exposure indicated that UV provided high energy as microwaves to cross-link proteins and AFB cells to the glass slide surface. We found the excellent fixation effect was also associated with BSA. However, MGIT or distilled water with BSA on the slides without UV exposure did not present the same object holding effect during staining. We therefore concluded that AFB cells adherence will be optimized by BSA and the high energy activation of UV light.

Microscopic visualization of AFB cell in the culture positive MGIT broth is the critical step for rapidly indicating the presence of mycobacteria. The earlier the AFB cell is detected is better, in terms of initiating an appropriate therapy and infection control of tuberculosis. In our study, there are less negative and scanty AFP results using modified (+UV) process than conventional (−UV) process. The earlier warning notification of these specimens will speed the control of tuberculosis. Although the slides were better prepared for observation by dark red staining with carbolfuchsin-BSA, masking effect by either darker staining or concomitantly enhanced attachment of cell debris may cause falsely negative microscopic observation potentially. However, the color and morphology of AFB positive cells and contaminated microbes on slide were less influenced by the dark red background. The observers were still able to easily distinguish AFB positive cells from contaminated microbes under the dark red background. All medical technicians involved in this study felt greater confidence on reporting AFB results even there were more contaminated bacteria and cell debris retained on slides. We did not intentionally evaluate whether the darker red background resulted in falsely positive observation of AFB positive cells in the MGIT(−) bottles, because they will neither be checked routinely in clinical practice. False positive observation of AFB positive cells was therefore not evaluated in this study. In conclusion, the findings of this study demonstrate the superior fixation effect of the modified fixation process. The modified fixation process can be easily utilized and of tremendous value in improving microscopic observation for AFB smears in a tuberculosis diagnosis laboratory.
